# The association of economic difficulties with social and health care costs of children—target trial emulation using complete birth cohort data in Finland

**DOI:** 10.1093/eurpub/ckae140

**Published:** 2024-09-17

**Authors:** Aapo Hiilamo, Markus Keski-Säntti, Aapo Juutinen, Lauri Mäkinen, Tiina Ristikari, Tea Lallukka

**Affiliations:** Laboratory of Population Health, Max Planck Institute for Demographic Research, Rostock, Germany; Max Planck—University of Helsinki Center for Social Inequalities in Population Health, Rostock, Germany; Department of Data and Analytics, Finnish Institute for Health and Welfare, Helsinki, Finland; Department of Public Health, Finnish Institute for Health and Welfare, Helsinki, Finland; Itla Children’s Foundation, Helsinki, Finland; Itla Children’s Foundation, Helsinki, Finland; Department of Public Health, Faculty of Medicine, University of Helsinki, Helsinki, Finland

## Abstract

It is unclear how much costs economic difficulties in families with children incur to the health and social care sector. We examined the health and social service costs after families entered into, and transitioned out of, social assistance used as a proxy measure for economic difficulties. We analyzed register data on all Finnish children born in 1997 and used the non-randomized target trial framework. The two target trials of entry to economic difficulties (social assistance) and continued economic difficulties included 697 680 and 71 131 children-year observations, respectively, in total. Inverse probability treatment weighting techniques were used to make the comparison group similar to the treatment group in terms of health, socioeconomic and demographic-related pretreatment variables. Entry to social assistance use was associated with some 1511–2619€ (50% compared to the control group) higher cumulative health and social care costs of the children three years after their families transitioned to social assistance, compared to the group that did not enter to social assistance system. This difference was primarily attributed to higher social care costs. Continued social assistance use was associated with some 1007–2709€ (31%) higher costs compared to the comparison group that exited social assistance. These findings support an economic argument to prevent families from entering economic difficulties and to help those in such situations to transition out.

Key points

- The transition to economic difficulties was associated with a substantial increase in health and social care costs per child.
- A considerable proportion of these costs were due to social care
- This evidence supports efforts to reduce economic difficulties in families as a public health and public saving strategy

## Introduction

In 2021, one-fourth of all children in the EU were at risk of poverty or social exclusion, while the rates varied significantly among the member countries [1]. The cost-of-living crises that emerged in 2022 have likely exacerbated the economic difficulties faced by families with children [2]. Child poverty is associated with poorer health outcomes and an increased need for social services [3], making the reduction of economic difficulties in families with children a public priority.

A systematic review of 54 quasi-experimental studies concluded that child poverty has a robust association with a range of negative outcomes [3]. Economic difficulties are linked to an increased risk of child mental health problems [4–6], poorer physical health [7], maltreatment [8, 9], chronic stress [10], and behavioral problems [11, 12]. Exogenous changes in economic circumstances, such as social policy programs [13, 14] or economic recessions [15], link also to changes in these outcomes, supporting the claim that a causal link exists between child poverty and negative outcomes. However, we did not identify any observational studies that used an individual level register data to assess the effects of childhood economic difficulties on public health and social care costs.

In this study, we examine the health and social service costs after families entered into, and transitioned out of, social assistance used as a proxy measure for economic difficulties. We use the target trial framework. Target trial emulation is a conceptual tool to help researchers to formulate their research questions and parameters of interest when randomized trials are not feasible due to practical or ethical concerns. In this framework an ideal trial is first conceptualized by defining the treatment and comparison groups, the start of the idealized trial and its follow-up period and the outcomes of interest [16, 17]. We use social assistance recipiency as a proxy measure for economic difficulties. We focus on the cost of children in the families transitioning to social assistance.

## Methods

We use the Finnish birth cohort data of 1997 (FBC 1997). FBC 1997 is a cohort study consisting of all children born in Finland in 1997. All individuals born in Finland in 1997 were identified from the national birth register and linked to their parents. Both parents and their children were linked to a range of administrative register data using the personal identification numbers. The dataset and its key findings are elaborated in previous studies [18]. The ethical approval for the use of this dataset has been obtained from the Ethics Committee of the Finnish Institute for Health and Welfare, THL (§572/2013). For the study at hand, we use register data drawn from the healthcare treatment notification register maintained by the THL, the population register by the Digital and Population Data Services Agency, the Social assistance register, and educational registers managed by Statistics Finland, among others.

The original birth cohort consisted of 58 802 children and their parents (mother *n* = 57 888 and father *n* = 57 222), from which we excluded individuals who moved abroad, deceased and children not living with their parents. Furthermore, we dropped randomly all but one of the siblings who had the same parents. Our unit of analysis is children-year while we measure a number of variables at the child and family levels. The same individual could be included multiple times in the analysis because we stacked all children-years together. The two target trials (see details below) had 54 858 and 16 026 cohort members including 697 680 and 71 131 children-year observations in total.

### Economic difficulties

Our variable of interest is a change in the recipiency of social assistance of the family in which the children lived. Social assistance is granted and measured at the family level. The data are derived from the national social assistance register. Data on social assistance recipiency was originally gathered from local municipalities and is managed by the Finnish Institute for Health and Welfare. We define social assistance recipiency as any situation where children’s parents had received social assistance at least once within the given calendar year. When a parent did not reside with their children (e.g. due to a separation), their social assistance records are not considered in our analysis.

In our study, social assistance is not treated as an instrument through which families’ economic difficulties are alleviated but as a proxy measure for the experienced economic difficulties. This is in line with our previous research with the same birth cohort data [19, 20]. The use of social assistance as proxy measure for economic difficulties is grounded on the fact that social assistance is intended for all households in Finland who have no spare income or assets to cover some basic living costs. For instance, a single adult was entitled to approximately 555 euros monthly in 2023. Other costs such as housing, medical care, and certain essentials are covered on a means-tested evaluation. Some tenth of children in Finland lives in a household that has received social assistance at least once in 2021 [21].

### Outcome

Our outcome variable of interest is the costs of specialized health care, social care and prescribed medication reimbursements in 2018 prices. In calculating the costs of specialized medical care, we used the Nordic Diagnosis Related Groups classification (NordDRG). NordDRG is a patient classification system used in Nordic countries that can be used to estimate the costs of specialized medical care [22]. Separate cost estimates have been made for psychiatric specialties using appropriate daily prices extrapolated from the THL's reports on the Unit Costs of Health and Social Care in Finland in 2001 [23] and Unit Costs of Health and Social Care in Finland in 2011 [24]. This is because the NordDRG classification is not well suited for estimating the costs of psychiatric specialties.

Costs in social care were calculated based on the daily rates of out-of-home care extrapolated from the report on the comparison of child protection services and costs in the six largest cities in Finland in 2015 [25]. Data on social care costs are based on the Register of Child Welfare [26]. Register of Child Welfare includes data from the reasons and duration of placement outside the home and the social care costs can be estimated by multiplying the duration by the appropriate daily rate. The Register of Child Welfare does not include information about open care and therefore these costs are not included in our analysis.

Medicine reimbursement costs are based on the defined daily dose (DDD), which is a measure of drug consumption. Data on medication reimbursements are from the registers of the Social Insurance Institution of Finland. Our data includes only psycholeptic and psychoanaleptic drugs (ATC: N05A–N06C). Costs of medicine reimbursement are estimated by multiplying the DDD included in the register by an average cost of one DDD extrapolated from the Finnish Statistics on Medicines [27].

### Control variables

To control for confounding, we derived health, socioeconomic and demographic variables from administrative registers that were measured before the treatment (time *t*−1). In our probability of treatment model, we included parental health status, measured using records of specialized health care records (4 variables), unemployment status (2 variables), highest educational attainment (2 variables), and earned income, transformed logarithmically for both the mother and father. Dichotomous variables accounting for no (recorded) earned income were included to measure extreme financial difficulties. We included variables measuring previous cumulative time spent in social assistance in months. We included cohort members’ sex, birth weight, imputed birth weight, and current values of social, psychiatric, and home care costs. We also calculated cumulative sums of these costs before the treatment and transformed them logarithmically.

### The definitions of the target trials

We use the target trial framework [16]. Target trials are ideal type of randomized controlled trials to be mimicked with observational data. We defined two types of target trials, which provide us with an analysis plan, i.e. selecting the sample, defining the treatment and comparison groups, and controlling for covariates.

The first we label as the “transition to social assistance use” trial. The population in this trial is all children in families not receiving social assistance in a given calendar year (*t*). The intervention group includes children in families that transition to receiving social assistance in the subsequent year (*t* + 1). The comparison group consists of the children with similar characteristics but in a scenario in which their families continue without social assistance in the following year (*t* + 1). The outcome of the trial is the health and social care costs incurred for these children 2–4 years later. This trial estimates the average treatment effect on the treated (ATT) of social assistance transition. This is that we contrast the social and health care costs in in the real-world scenario with a counterfactual scenario in which these same families did not transition into social assistance. This trial provides information about the potential savings if the transition into social assistance usage could be completely prevented.

In our second target trial, labelled as “continued social assistance use,” the population consists of children in families receiving social assistance in a given calendar year (*t*). The intervention is defined as families receiving social assistance also in the subsequent year (*t* + 1). This group is compared with the same families but in a scenario in which they did not receive social assistance in the following year, i.e. transitioned out from social assistance use (*t* + 1). The outcomes are identical to the first trial. In other words, in this trial we are compare the costs between the actual scenario, where these families continued to receive social assistance and a hypothetical scenario where families did transition out from social assistance. This target trial quantifies the potential savings in the costs that could result if the families that were unsuccessful in transitioning out from social assistance would have been successful.

We conducted these trials for three populations; first, all children (all years pooled, same children could be contributing many times), to test moderation by age, children aged 0–7 before the change in social assistance status (years 1998–2004 pooled) and children aged 8–14 (2004–11).

### Statistical analysis

To make the comparison group similar to the treatment group, we used inverse probability treatment weighting. We start by estimating the probability of treatment models in which we predicted the probability of social assistance status at *t* + 1 separately for both trial. We fit logistic regression models in which we regress the social assistance status at time *t* + 1 on the covariates listed above. We fit these models by year to allow all interactions with year. From the model, we then calculate the propensity scores (ps) and set the inverse probability of treatment weights (IPTW) to 1 if the observation was in the treatment group (received social assistance in the subsequent year) and as ps / (1−ps) if the observation was in the comparison group (did not receive social assistance in the subsequent year) [28]. We then calculated the IPTW weighted averages of the costs in the social and health care registers in the following calendar years from the social assistance transition for the full sample. We present the balance statistics as recommended in the Supplementary Materials [28]. To assess the uncertainty of our estimates, we use the bootstrapping method and calculate 95 percentile intervals from 500 replications. Our estimation assumes no misspecifications in the treatment model and no unmeasured confounding over and above the dimensions captured by the measured control variables [29]. R software version 4.1.1. was used in all phases of the analysis.

## Results

The analysis investigating the transition to social assistance use consisted of 680 314 observations in the comparison group (did not transition to social assistance) and 17 366 in the treatment groups (transitioned to social assistance use). The children in the treated group had a lower parental education level, more parental health records, a higher share of parental unemployment, and lower parental income compared to the comparison group who did not transition to social assistance (Table 1). Previous social and health care costs of children in the treatment group were also higher. However, IPTW balanced these differences, and few variables had standardized differences between the groups higher than 0.1 (see online Supplementary Material for a colour version of this Supplementary Fig. S1).

**Table 1.** Characteristics of the study population in the social assistance entry trial

	All children		Children aged 0–7		Children aged 8–14	
	Comparison	Treated	Comparison	Treated	Comparison	Treated
Mother’s education						
At least upper university level	13.46%	1.74%	12.8%	1.7%	14.07%	1.79%
Lower university level	36.85%	16.28%	37.01%	17.04%	36.71%	15.22%
Middle level	40.42%	52.95%	40.15%	51.23%	40.68%	55.37%
Basic level	9.27%	29.03%	10.04%	30.03%	8.54%	27.62%
Father’s education level						
At least upper university level	12.31%	2.08%	12.16%	1.93%	12.46%	2.29%
Lower university level	25.48%	10.8%	25.72%	10.66%	25.26%	10.99%
Middle level	46.55%	53.28%	46.47%	53.46%	46.62%	53.03%
Basic level	15.65%	33.84%	15.65%	33.95%	15.66%	33.69%
Mother’s somatic illness	21.51%	27.48%	19.23%	24.52%	23.65%	31.65%
Father’s somatic illness	18.87%	23.56%	16.86%	21.45%	20.75%	26.52%
Father’s unemployment	6.87%	19.16%	2.78%	13.56%	10.7%	27.03%
Mother’s unemployment	9.82%	25.75%	4.94%	16.41%	14.4%	38.9%
Father’s psychiatric illness	1.19%	3.9%	0.83%	3.15%	1.53%	4.95%
Mother’s psychiatric illness	1.43%	4.49%	0.99%	3.31%	1.85%	6.16%
Birth weight imputed^a^	0.22%	0.12%	0.22%	0.13%	0.22%	0.1%
Cumulative somatic health care costs	3960.62	4554.73	2918.99	3390.64	4936.05	6193.32
Cumulative psychiatric health care costs	316.14	628.66	109.15	173.08	509.97	1269.93
Cumulative social care costs	210.16	1448.88	58.53	467	352.14	2830.97
Father’s earned income (log)	9.01	7.61	8.47	7.58	9.51	7.66
Mother’s earned income (log)	8.09	5.72	6.97	4.86	9.15	6.91
No earned income father	0.13%	0.23%	0.18%	0.23%	0.09%	0.23%
No earned income mother	0.18%	0.38%	0.27%	0.46%	0.09%	0.27%
Previous time in social assistance mother (months)	0.52	2.73	0.28	1.59	0.75	4.35
Previous time in social assistance father (months)	0.5	2.37	0.26	1.43	0.72	3.68
Birth weight	3558.92	3521.38	3560.57	3523.04	3557.39	3519.05
Social care costs before treatment	36.9	215.22	9.75	73.81	62.33	414.26
Psychiatric health care costs before treatment	61.52	159.79	33.77	65.46	87.5	292.57
Somatic health care costs before treatment	346.22	472.27	429.58	562.67	268.15	345.03
Medicine costs before treatment	1.67	3.06	0.05	0.06	3.19	7.28
*n*	680 314	173 66	328 992	10 153	351 322	7213

a This is binary variable that birth weight data was not available and imputed for the mean of the sample.

Treated group are children in families entering to social assistance use. The comparison group are children in families not entering to social assistance use. The FBC 1997.

After the IPTW balancing, the two groups had little difference in the costs in the year before the transition (a *t*−1 difference of −23€ [bootstrap 95% percentile interval of −431 to 32]) but had significant differences a year (608€ [406–762]), 2 years (742€ [510–943]), and 3 years after the transition (742€ [507–997], Table 2). The cumulative three-year difference was 2092€ per child (1511–2619€). This is that the treatment group had some 50% higher costs than the comparison group. This difference was due to higher social care costs (1794€ [1276–2260] difference, 72% higher than the comparison group). Smaller differences were observed in somatic health care costs (151€ [36–263] 14% difference) and in psychiatric health care costs (146€ [4–316], 23% difference). Age-stratified analysis suggested that the reduction was stronger in absolute terms in the 8–14 aged population (a 3372€ [2108–4405] difference) than in the 0–7 aged population (a 1185€ [774–1599] difference).

**Table 2.** Costs estimates in Euros for the social assistance entry trial, estimated via inverse probability treatment weighting

	ALL children		Children aged 0–7 (aged 1–7)		Children aged 8–14 (8–14)	
	ATT (95% bootstrap percentile)	ATT/comparison mean	ATT (95% bootstrap percentile)	ATT/comparison mean	ATT (95% bootstrap percentile)	ATT/comparison mean
All costs						
T−1	−23 [−431 to 32]	−3%	−29 [−532 to 20]	−4%	−15 [−379 to 97]	−1%
T + 1	608 [406–762]	56%	421 [244–570]	70%	873 [499–1196]	49%
T + 2	742 [510–943]	52%	403 [243–564]	60%	1221 [763–1648]	49%
T + 3	742 [507–997]	44%	361 [184–540]	46%	1279 [733–1749]	43%
Sum t1−t3	2092 [1511–2619]	50%	1185 [774–1599]	58%	3372 [2108–4405]	46%
Health care costs—somatic						
T−1	−13 [−395 to 9]	−3%	−22 [−487 to 11]	−4%	0 [−84 to 22]	0%
T + 1	66 [4–114]	18%	112 [21–190]	30%	1 [−43 to 33]	0%
T + 2	56 [8–107]	16%	76 [12–148]	22%	28 [−18 to 79]	7%
T + 3	29 [−6 to 64]	8%	56 [8–106]	17%	−10 [−48 to 31]	−2%
Sum *t*1−*t*3	151 [36–263]	14%	244 [54–405]	23%	19 [−75 to 110]	2%
Health care costs—psychiatric						
T−1	6 [−47 to 43]	4%	−2 [−13 to 5]	−3%	19 [−249 to 101]	7%
T + 1	47 [−26 to 113]	24%	−12 [−91 to 42]	−10%	130 [10–289]	42%
T + 2	62 [−1 to 134]	30%	5 [−46 to 47]	5%	143 [−3 to 309]	41%
T + 3	38 [−44 to 157]	17%	0 [−37 to 28]	0%	91 [−112 to 323]	24%
Sum *t*1−*t*3	146 [4–316]	23%	−8 [−110 to 82]	−2%	364 [16–702]	35%
Social care costs						
T−1	−17 [−85 to 16]	−7%	−5 [−74 to 25]	−6%	−34 [−114 to 28]	−8%
T + 1	496 [335–621]	96%	321 [205–418]	300%	742 [420–1016]	68%
T + 2	624 [430–794]	73%	322 [196–439]	147%	1049 [630–1423]	60%
T + 3	675 [456–878]	61%	305 [159–463]	91%	1196 [756–1641]	54%
Sum *t*1−*t*3	1794 [1276–2260]	72%	948 [610–1266]	143%	2987 [1866–3970]	59%
Medical reimbursement costs						
T−1	0 [0–0]	0%	0 [0–0]	0%	0 [0–1]	0%
T + 1	0 [−1 to 1]	0%	0 [0–0]	0%	1 [−1 to 3]	8%
T + 2	0 [−1 to 1]	0%	0 [−1 to 0]	0%	1 [−2 to 3]	7%
T + 3	1 [−1 to 2]	12%	0 [−1 to 0]	0%	2 [−2 to 4]	12%
Sum *t*1−*t*3	1 [−2 to 4]	5%	0 [−2 to 1]	0%	3 [−4 to 9]	7%

ATT is the average treatment effect on the treated. Treated group are children in families entering to social assistance use. The comparison group are children in families not entering to social assistance use. Observations = 697 680. The FBC of 1997

The second type of analysis, concerning continued social assistance use, consisted of 22 971 observations in the comparison group (transitioned out of) and 48 160 in the treatment group (continued to use social assistance). The treated group had lower income and longer periods of previous social assistance use than the comparison group that exited social assistance (Table 3). After the IPTW balancing, there were no significant differences in the costs before the treatment (37 [−176 to 194] difference, Table 4.). The continued social assistance recipiency group had 518 [130–774] higher costs 1 year, 636 [302–913] 2 years, and 820 [475–1136] 3 years after the treatment. This resulted in a cumulative difference of 1974 [1007–2709], compared to the comparison group and 31% higher costs. This difference was driven by differences in social care costs (1909 [977–2608], 43%). Minor differences were observed in somatic health care costs (146 [74–216] €, 13%). No significant differences were observed in psychiatric healthcare costs. No clear age group differences were observed due to the uncertainty of the estimates.

**Table 3.** Characteristics of the study population in the continued social assistance trial

	ALL children		Children aged 0–7		Children aged 8–14	
	Comparison	Treated	Comparison	Treated	Comparison	Treated
Mother’s education						
At least upper university level	1.96%	0.98%	2.01%	0.85%	1.86%	1.24%
Lower university level	17.07%	9.08%	17.96%	9.17%	15.37%	8.91%
Middle level	52.96%	45.01%	51.31%	43.42%	56.11%	48.04%
Basic level	28.01%	44.92%	28.72%	46.56%	26.66%	41.81%
Father’s education level						
At least upper university level	2.14%	1.19%	2.14%	1.01%	2.15%	1.53%
Lower university level	10.98%	7.6%	11.18%	7.19%	10.6%	8.39%
Middle level	52.67%	46.2%	52.45%	45.98%	53.09%	46.62%
Basic level	34.21%	45%	34.23%	45.82%	34.17%	43.46%
Mother’s somatic illness	26.37%	28.41%	23.36%	25.45%	32.1%	34.01%
Father’s somatic illness	23.35%	25.2%	21.39%	23.71%	27.08%	28.01%
Father’s unemployment	26.72%	42.95%	22.96%	42.4%	33.85%	43.98%
Mother’s unemployment	32.3%	39.17%	22.96%	29.46%	50.06%	57.53%
Father’s psychiatric illness	4.62%	5.77%	3.71%	5.16%	6.34%	6.92%
Mother’s psychiatric illness	4.59%	6.1%	3.29%	4.44%	7.05%	9.25%
Birth weight imputed*	0.11%	0.23%	0.13%	0.2%	0.09%	0.28%
Cumulative somatic health care costs	4349.74	4858.36	3377.96	3739.01	6197.55	6973.05
Cumulative psychiatric health care costs	519.91	647.44	161.68	188.12	1201.09	1515.19
Cumulative social care costs	1586.3	3245.35	638.53	1522.96	3388.46	6499.29
Father’s earned income (log)	7.45	5.73	7.45	5.72	7.46	5.75
Mother’s earned income (log)	5.57	3.45	4.97	2.9	6.72	4.5
No earned income father	0.23%	0.39%	0.23%	0.38%	0.24%	0.4%
No earned income mother	0.38%	0.6%	0.43%	0.65%	0.27%	0.49%
Previous time in social assistance mother (months)	4.09	5.37	3.01	3.74	6.12	8.46
Previous time in social assistance father (months)	3.22	4.45	2.45	3.21	4.67	6.79
Birth weight	3515.53	3473.79	3516.48	3473.53	3513.73	3474.27
Social care costs before treatment	318.11	706.33	175.37	428.58	589.55	1231.06
Psychiatric health care costs before treatment	123.88	151.86	55.98	65.63	252.98	314.78
Somatic health care costs before treatment	529.59	575.18	627.25	674.76	343.9	387.04
Medicine costs before treatment	2.39	3.34	0.08	0.09	6.78	9.5
*n*	22 971	48 160	15 054	31 491	7917	16 669

Treated group are children in families with continued social assistance use. The comparison group are children in families exiting social assistance use. The FBC 1997.

**Table 4.** Costs estimates in Euros for the continued social assistance use trial, estimated via inverse probability treatment weighting

	ALL children		Children aged 0–7		Children aged 8–14	
	ATT (95% bootstrap percentile to be added)	Treatment/comparison mean	ATT (95% bootstrap percentile to be added)	Treatment/comparison mean	ATT (95% bootstrap percentile to be added)	Treatment/comparison mean
All costs						
T−1	37 [−176 to 194]	3%	19 [−151 to 160]	2%	67 [−703 to 374]	4%
T + 1	518 [130–774]	31%	475 [270–689]	49%	589 [−841 to 1211]	19%
T + 2	636 [302–913]	29%	579 [297–833]	47%	728 [−419 to 1391]	18%
T + 3	820 [475–1136]	33%	723 [413–1000]	50%	985 [−140 to 1691]	22%
Sum *t*1−*t*3	1974 [1007–2709]	31%	1778 [1043–2441]	49%	2302 [−1058 to 4098]	20%
Health care costs—somatic						
T−1	25 [−4 to 61]	5%	27 [−32 to 67]	4%	21 [−24 to 69]	6%
T + 1	72 [38–108]	19%	86 [41–128]	23%	46 [−10 to 96]	13%
T + 2	54 [24–86]	14%	55 [12–97]	15%	51 [0–107]	13%
T + 3	20 [−17 to 55]	5%	38 [−2 to 71]	11%	−14 [−104 to 70]	−3%
Sum *t*1−*t*3	146 [74–216]	13%	180 [78–269]	17%	83 [−42 to 214]	7%
Health care costs—psychiatric						
T−1	−5 [−43 to 30]	−3%	−6 [−25 to 6]	−8%	−4 [−157 to 88]	−1%
T + 1	−49 [−223 to 42]	−19%	3 [−23 to 26]	3%	−151 [−601 to 117]	−28%
T + 2	−28 [−110 to 40]	−11%	−2 [−36 to 32]	−1%	−78 [−306 to 106]	−17%
T + 3	−3 [−62 to 48]	−1%	26 [−6 to 54]	19%	−60 [−202 to 78]	−14%
Sum *t*1−*t*3	−80 [−280 to 82]	−11%	28 [−42 to 94]	7%	−290 [−812 to 136]	−21%
Social care costs						
T−1	17 [−191 to 151]	2%	−3 [−145 to 134]	−1%	50 [−595 to 307]	4%
T + 1	496 [204–716]	47%	385 [179–596]	80%	695 [−514 to 1189]	33%
T + 2	611 [279–868]	39%	527 [263–764]	74%	757 [−369 to 1415]	24%
T + 3	802 [476–1102]	44%	659 [368–926]	68%	1059 [−39 to 1687]	30%
Sum *t*1−*t*3	1909 [977–2608]	43%	1571 [863–2254]	73%	2511 [−798 to 4233]	29%
Medical reimbursement costs						
T−1	0 [−2 to 1]	0%	0 [0–0]	0%	−1 [−7 to 2]	−10%
T + 1	0 [−3 to 1]	0%	0 [0–0]	0%	−1 [−12 to 3]	−7%
T + 2	−1 [−4 to 1]	−14%	0 [−1 to 0]	0%	−1 [−14 to 4]	−6%
T + 3	0 [−3 to 2]	0%	0 [−1 to 1]	0%	0 [−10 to 5]	0%
Sum *t*1−*t*3	−1 [−10 to 4]	−5%	0 [−2 to 1]	0%	−2 [−36 to 11]	−4%

ATT is the average treatment effect on the treated. Treated group are children in families with continued social assistance use. The comparison group are children in families exiting social assistance use. Observations = 71 131. The FBC of 1997.

## Discussion

This study is, to the best of our knowledge, the first population-based attempt to quantify the health and social care costs of children due to economic difficulties in their families using a target trial framework. Our results suggest substantial costs saving potential of preventing economic difficulties in families with children.

The transition to social assistance, a proxy for economic difficulties in families with children, was associated with a substantial increase in health and social care costs per child over a 3-year period. A considerable proportion of these costs were due to social care while significant differences were observed for health care costs. This finding is in line with the long line of research showing an association of entry into economic difficulties with health and development problems of children [3]. For example, an UK cohort study showed that transition into income poverty increased risk of child mental health problems [4].

The second part of the analyses focused on the effects of continued social assistance use vs. exiting social assistance use. We found that children in families that continued in social assistance had 31% higher costs than the comparison group. This difference was again driven by social care costs. We did not find significant differences in psychiatric health care costs in this part of the analysis. This finding may be related to lasting effects of economic difficulties in families.

Some previous estimates suggest that economic burden of child poverty are to a substantial extent due to increased health care costs [30–32] but our results indicate that the main cost concern is social care. This is not surprising considering the accumulated evidence on the link between poverty and child welfare intervention needs. For example, a previous ecological analysis link social assistance uses to a higher rate of out-of-home care in Finnish municipalities [33].

We did not investigate the mechanism through which economic difficulties link to higher costs. Previous studies suggest mechanisms such as increasing stress, mental health problems, family instability, and substance abuse, all of which may affect the need for health and social services. Stress may arise from parental economic difficulties which may trigger physical and mental health issues. Economic difficulties may link to stigma, bullying, social exclusion, fewer friends, and constrained opportunities for past time activities. For these discussions see Ref. [5]. An important mechanism here may also be that social assistance use increases the likelihood of being in contact with social workers and more so when the social assistance use is prolonged. During the time of this study period there was strong connection between social work and social assistance because social assistance was granted by the municipalities. More frequent contact with social workers may increase the likelihood of severe, and more expensive, child welfare measures. Our study does not provide us answer whether the social care costs increased due to changes in the family *per se* or due to changes in the likelihood of being assigned to child welfare measures.

There are several limitations in this study. There are more than one way of transitioning to social assistance or to continue receiving social assistance. Thus, our estimates are to be conceptualized as an unknown weighted average of the various ways in which families transition to social assistance, compared to unknown weighted average of the different ways in which families do not change their social assistance status. We stacked all the children-year observations together to increase statistical power, resulting a mix of different ages at which children’s families entered to social assistance.

Our analyses used non-randomized observational data and the estimates presented here are subject to unmeasured confounding. The unadjusted models showed stronger effects, suggesting that the role of confounding is important. While we included exceptionally high number of control variables, some residual confounding certainly remains. Moreover, we used social assistance as a proxy for economic difficulties, which may not always reflect the economic difficulties experienced by families. On the one hand, those experiencing economic difficulties may not apply for social assistance due to stigma or bureaucratic burden. There is no comparable data on families with children but it has been suggested that some 30%–50% of the eligible population do not receiving social assistance [34]. Families with children may also experience financial difficulties without being eligible for social assistance use, e.g. due to debt repayments. On the other hand, those receiving only temporary social assistance may not experience severe economic difficulties if social assistance is used to bridge over temporal economic difficulties, such as short-term unemployment.

Several brackets of costs that were not included in our study. First, we did not have data on primary health care. This implies that the health care costs here are underestimated. Second, we lacked information on health care costs of the parents or other family members. Third, we did not make any cost-calculations of specialized services provided in schools. Fourth, we did not take into consideration potential costs in the criminal correction system.

Nevertheless, these results add to the evidence supporting efforts to reduce economic difficulties in families as a public health and public saving strategy. This is particularly the case regarding social care costs, which could be potentially reduced by preventing social assistance use. More preventative measures are needed such as improving employment security and the level of universal social benefits to prevent families with children entering social assistance use.

## Conclusion

This is the first population-based investigation aiming to quantify the health and social care costs of children due to economic difficulties. There is substantial cost-saving potential in preventing economic difficulties in families with children. These findings add to increasing body of evidence that support efforts to reduce economic difficulties in families as a public health and public saving strategy.

## Supplementary Material

ckae140_Supplementary_Data

## Data Availability

The analyzed data are not publicly available due to strict privacy laws. Inquiries about the access to the data can be send to Markus Keski-Säntti.
