# Supplementary material for: The association of economic difficulties with social and health care costs of children—target trial emulation using complete birth cohort data in Finland
Source: Eur J Public Health. 2024 Sep 17;34(6):1036–42. doi: 10.1093/eurpub/ckae140 (PMC11631535; doi:10.1093/eurpub/ckae140)
Supplement: ckae140_Supplementary_Data [file ckae140_supplementary_data.docx]

**
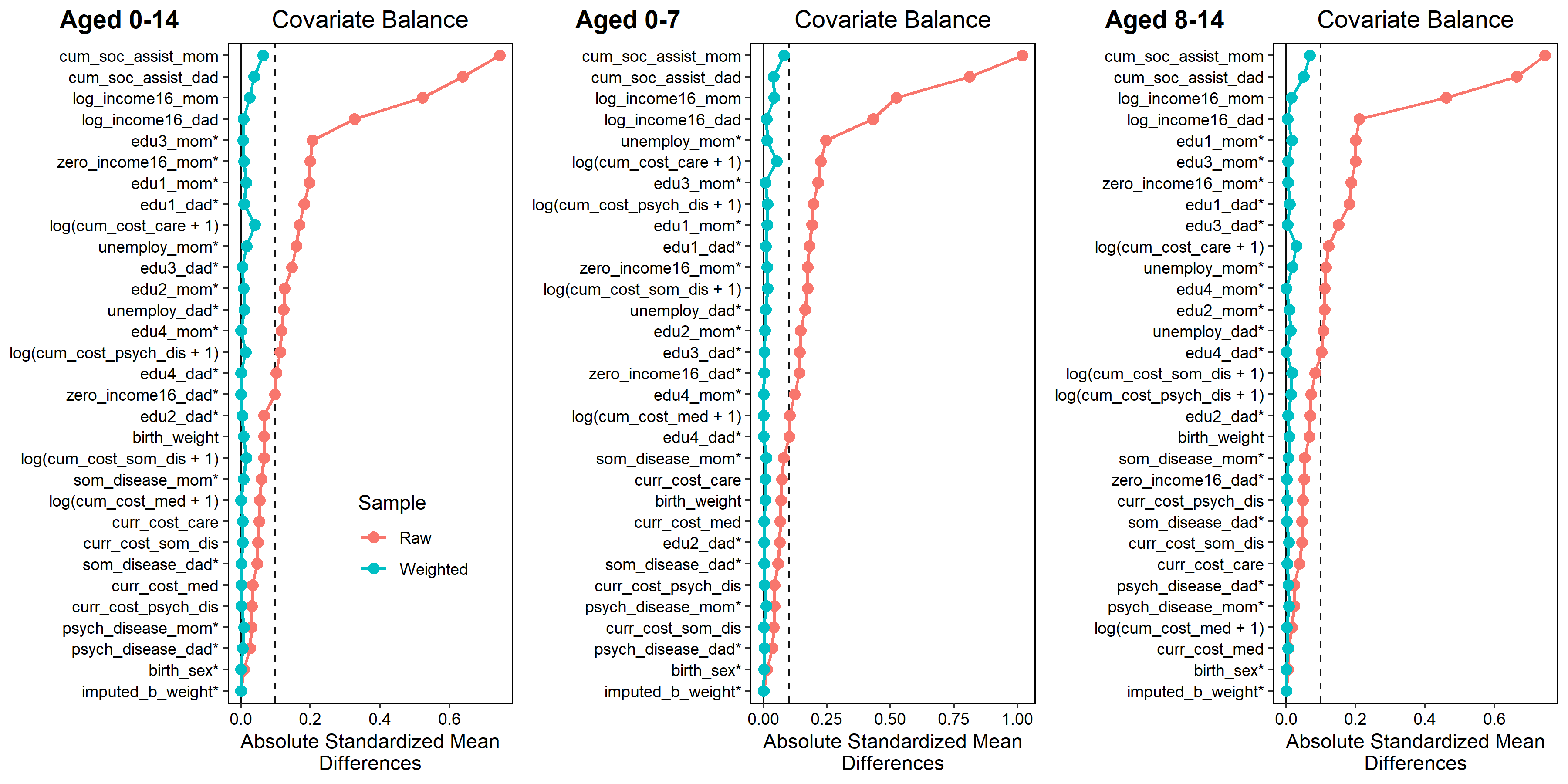
**

Supplementary Figure 1. Balance characteristics of the study population in the social assistance entry trial. Absolute mean differences before and after weighting. observations = 697,680 The Finnish Birth Cohort 1997.

**
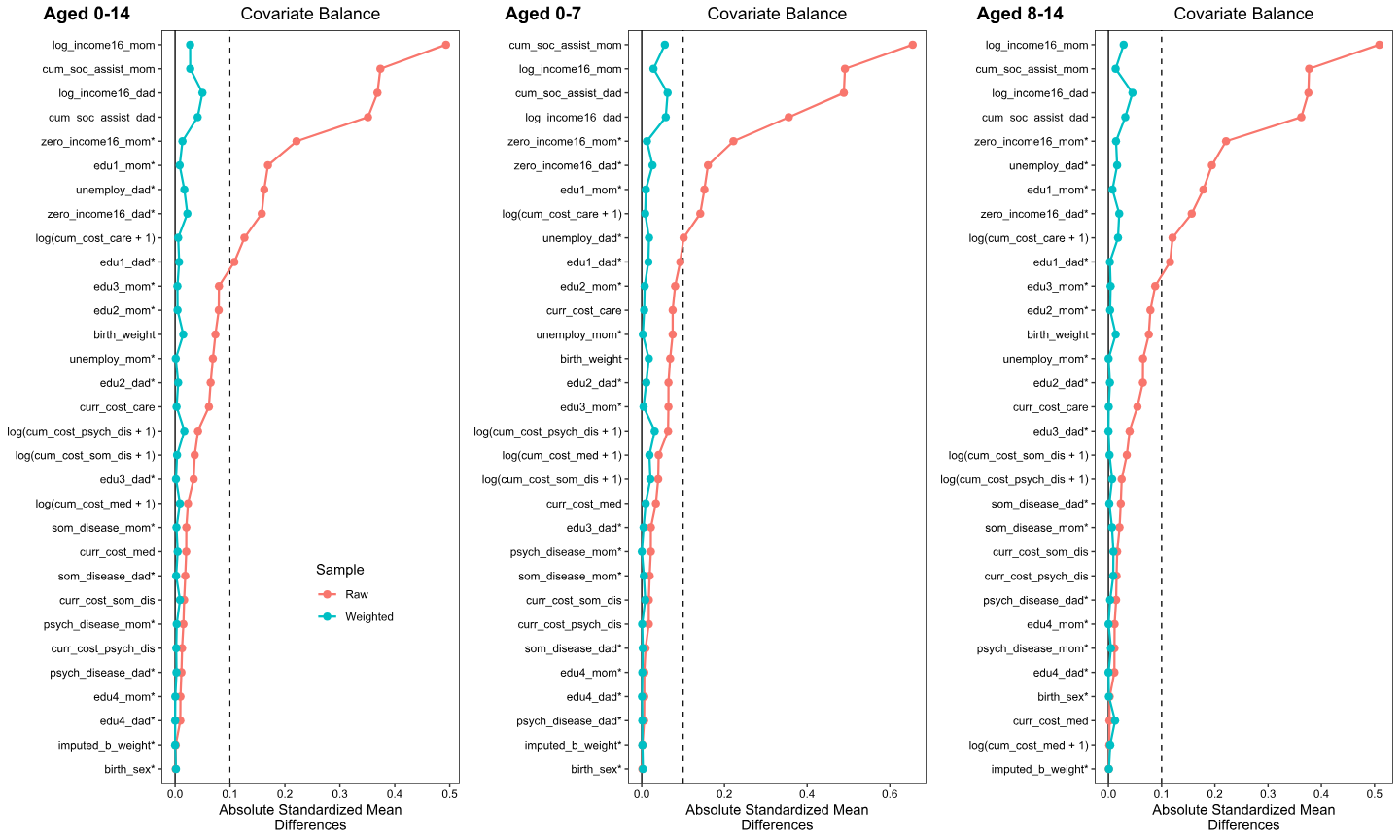
**

Supplementary Figure 2. Balance characteristics of the study population in the continued social assistance entry trial. Absolute mean differences before and after weighting. Observations = 71 131. The Finnish Birth Cohort 1997.
